# Threonyl-tRNA synthetase overexpression correlates with angiogenic markers and progression of human ovarian cancer

**DOI:** 10.1186/1471-2407-14-620

**Published:** 2014-08-27

**Authors:** Theresa L Wellman, Midori Eckenstein, Cheung Wong, Mercedes Rincon, Takamaru Ashikaga, Sharon L Mount, Christopher S Francklyn, Karen M Lounsbury

**Affiliations:** Departments of Pharmacology, University of Vermont, College of Medicine, Burlington, Vermont 05405 USA; Departments of Obstetrics, Gynecology and Reproductive Sciences, Burlington, Vermont 05405 USA; Departments of Medicine, University of Vermont, College of Medicine, Burlington, Vermont 05405 USA; Medical Biostatistics, University of Vermont, College of Medicine, Burlington, Vermont 05405 USA; Departments of Pathology, University of Vermont, College of Medicine, Burlington, Vermont 05405 USA; Departments of Biochemistry, University of Vermont, College of Medicine, Burlington, Vermont 05405 USA; The Vermont Cancer Center, University of Vermont, College of Medicine, Burlington, Vermont 05405 USA

**Keywords:** Tumor microenvironment, Angiogenesis, tRNA synthetase, Serous papillary ovarian cancer, Database analysis, Multivariate Cox analysis

## Abstract

**Background:**

Ovarian tumors create a dynamic microenvironment that promotes angiogenesis and reduces immune responses. Our research has revealed that threonyl-tRNA synthetase (TARS) has an extracellular angiogenic activity separate from its function in protein synthesis. The objective of this study was to test the hypothesis that TARS expression in clinical samples correlates with angiogenic markers and ovarian cancer progression.

**Methods:**

Protein and mRNA databases were explored to correlate TARS expression with ovarian cancer. Serial sections of paraffin embedded ovarian tissues from 70 patients diagnosed with epithelial ovarian cancer and 12 control patients were assessed for expression of TARS, vascular endothelial growth factor (VEGF) and PECAM using immunohistochemistry. TARS secretion from SK-OV-3 human ovarian cancer cells was measured. Serum samples from 31 tissue-matched patients were analyzed by ELISA for TARS, CA-125, and tumor necrosis factor-α (TNF-α).

**Results:**

There was a strong association between the tumor expression of TARS and advancing stage of epithelial ovarian cancer (p < 0.001). TARS expression and localization were also correlated with VEGF (p < 0.001). A significant proportion of samples included heavy TARS staining of infiltrating leukocytes which also correlated with stage (p = 0.017). TARS was secreted by ovarian cancer cells, and patient serum TARS was related to tumor TARS and angiogenic markers, but did not achieve significance with respect to stage. Multivariate Cox proportional hazard models revealed a surprising inverse relationship between TARS expression and mortality risk in late stage disease (p = 0.062).

**Conclusions:**

TARS expression is increased in epithelial ovarian cancer and correlates with markers of angiogenic progression. These findings and the association of TARS with disease survival provide clinical validation that TARS is associated with angiogenesis in ovarian cancer. These results encourage further study of TARS as a regulator of the tumor microenvironment and possible target for diagnosis and/or treatment in ovarian cancer.

**Electronic supplementary material:**

The online version of this article (doi:10.1186/1471-2407-14-620) contains supplementary material, which is available to authorized users.

## Background

Although mortality rates have decreased for most gynecologic malignancies, ovarian cancer remains the most lethal gynecologic cancer and ranks fifth in cancer deaths among women [1]. Critical issues contributing to poor prognosis include the inability to detect early stage disease and the capacity of ovarian cancer cells to manipulate the tumor microenvironment [2–4]. The current serum biomarker cancer antigen-125 (CA-125) is not a reliable indicator of ovarian cancer in pre-menopausal women and its use in combination with other screening techniques has not improved survival [5].

Ovarian cancer is highly angiogenic, and our laboratory and others have correlated ovarian cancer progression with the expression of angiogenic signaling molecules, including hypoxia-inducible factor (HIF-1α) and vascular endothelial growth factor (VEGF) [6, 7]. A continuing theme in effective angiogenesis is the importance of secreted cytokines including tumor necrosis factor-α (TNF-α) that exert both autocrine and paracrine effects on inflammatory and vascular endothelial cells [8]. This interaction network of secreted factors holds important clues for future diagnostic and treatment targets. It is therefore critical to continue advances in understanding the underlying cell signaling associated with the developing ovarian cancer microenvironment.

The study of threonyl tRNA-synthetase (TARS) as an extracellular angiogenic factor was instigated by the observation that the TARS inhibitor borrelidin has an anti-angiogenic effect [9]. Although the canonical function of TARS is to charge threonine onto tRNA during protein synthesis, it has been identified as an auto-antibody (PL-7) target in myositis autoimmune disorders [10]. The etiology of these diseases includes TNF-α signaling, and there is an epidemiological linkage between myositis and several different cancers [11, 12]. Moreover, we recently discovered that TARS has direct extracellular angiogenic activity both *in vitro* and *in vivo* through a mechanism that includes attraction of endothelial cells [13]. These lines of evidence led to the hypothesis that TARS plays a role in the tumor microenvironment and may be an indicator of progression in angiogenic and/or inflammatory cancers.

Here we explored the relationship between TARS and human ovarian cancer. We provide the first report that levels of TARS in patient tumors and inflammatory cells correlate with angiogenesis and stage of disease. The secretion of TARS by ovarian cancer cells, its presence within patient serum, and the negative relationship between tumor TARS and mortality risk highlight the potential of TARS as a target in the clinical management of ovarian cancer.

## Methods

### Database analysis

The SAGE anatomic viewer within the Cancer Gene Anatomy Project database (CGAP) (http://cgap.nci.nih.gov/SAGE/Viewer?TAG=GCAGACATTG&CELL=0&ORG=Hs&METHOD=SS10,LS10) was used to assess mRNA expression levels of TARS within normal and malignant tissues. The Gene Expression Omnibus (GEO) database (http://www.ncbi.nlm.nih.gov/geoprofiles/40739453) was used to search existing mRNA profiles related to ovarian cancer patient studies [14]. The Human Protein Atlas (http://www.proteinatlas.org/ENSG00000113407/cancer) provided information about TARS and cancers within a set of immunostained tissue arrays [15].

### Ovarian patient study group

This research was approved by the University of Vermont’s institutional review board (CHRMS 00–260, 01–026, 12–004). Written informed consent for participation in the study was obtained from all patients. The study group consisted of 70 patients diagnosed with epithelial ovarian cancer at Fletcher Allen Health Care/University of Vermont between 1999 and 2003. The control group consisted of 12 women who underwent oophorectomies that were identified as benign pathologies (See Additional file 1: Table S1A). Ovarian tissue samples were fixed with formalin and embedded in paraffin. Histological subtype was according to the WHO classification and stage was determined by FIGO criteria. Blood samples were obtained from a subset of patients (6 control, 31 cancer) prior to surgery (See Additional file 1: Table S1B). Serum was prepared by centrifugation and then cryopreserved until use. Patient survival information was obtained using the Fletcher Allen electronic health record system (PRISM).

### Immunohistochemistry (IHC)

Serial sections (5 μm) from each paraffin-embedded specimen were cut, transferred to slides, and then analyzed using immunohistochemistry to measure the expression of TARS, VEGF and PECAM (CD31) as in Wong et al. [6]. Immunoperoxidase staining was performed using the following antibodies: monoclonal anti-TARS (1:100 Novus, NB H00006897-M01, clone 1A9), monoclonal anti-VEGF (1:100 Santa Cruz Biotechnology, SC-7269), and monoclonal anti-PECAM (1:40 anti-CD31, DAKO, M-0823). No primary antibody was used as a negative control. TARS antibody staining was optimized for the greatest range of detection by testing multiple dilutions (1:50–1:300) using benign and Stage 3 ovarian tumor sections. Secondary antibody was DAKO Polymer–HRP goat anti-mouse IgG. Cells were lightly counterstained with Mayers’ hematoxylin and slides were dehydrated and then mounted using Cytoseal-60. Images were obtained using an Olympus BX50 light microscope coupled to a CCD camera and Metamorph image capture software. For TARS and VEGF, images were scored blindly by 2 different investigators for expression level using a scale of 1–4, where 1 was no staining and 4 was intense staining.

### Cultured cell experiments

SK-OV-3 human ovarian cancer cells were cultured in McCoy’s media supplemented with 10% fetal bovine serum as described in [16]. For secretion experiments, cells were grown in 10 cm dishes and serum-starved for 24 h followed by treatment with 10 ng/ml TNF-α or exposure to 2% O_2_ for 16 h. Media was collected and concentrated 20-fold using Amicon® Ultra-4 centrifugal filters. Cell membrane integrity was confirmed using the lactate dehydrogenase assay CytoTox-ONE™. Cell lysates (5%) and corresponding concentrated cell media (25%) were separated by SDS-PAGE and analyzed by Western blot for presence of TARS (1:1000 rabbit-anti-ThrRS, Santa Cruz Biotechnology SC-98543) as described in [13]. β-tubulin was detected as a loading control for the lysates and to confirm that TARS found in the media was not a result of cell lysis.

To measure relative mRNA levels, SK-OV-3 cells were grown in 6-well dishes. After treatments, mRNA was extracted and purified using a Qiagen RNeasy® kit. Double stranded cDNA was generated and relative mRNA levels were quantified by RT-qPCR using Taqman® Assays on Demand™ for TARS, VEGF, and interleukin-1β (IL-1β) with β2 microglobulin as the reference gene (Applied Biosystems). Values were determined by the comparative CT method and presented as Rq (2^ΔΔCT^).

### ELISA

ELISA was performed on 50 μl of SK-OV-3 cell media or patient serum samples using commercially available reagents to measure human TARS (Cusabio Biotech) or TNF-α (R&D Systems Quantikine® HS ELISA). Values were determined by comparing to a standard curve (0–800 pg/ml for TARS, 0–32 pg/ml for TNF-α).

### Statistical analysis

Statistical analysis was performed using SYSTAT software. Significance between group averages was determined by one way ANOVA followed by pair-wise multiple comparisons. Equal variance within the datasets was confirmed by Bartlett Chi-square analysis. Relationships between variables were examined using Pearson correlations. Cox proportional hazard models were used to analyze survival outcomes. Multivariate Cox models were also generated to consider the effects of stage grouping and year of diagnosis on the outcome.

## Results

### Database analysis of TARS expression in ovarian cancer

**Figure 1:**
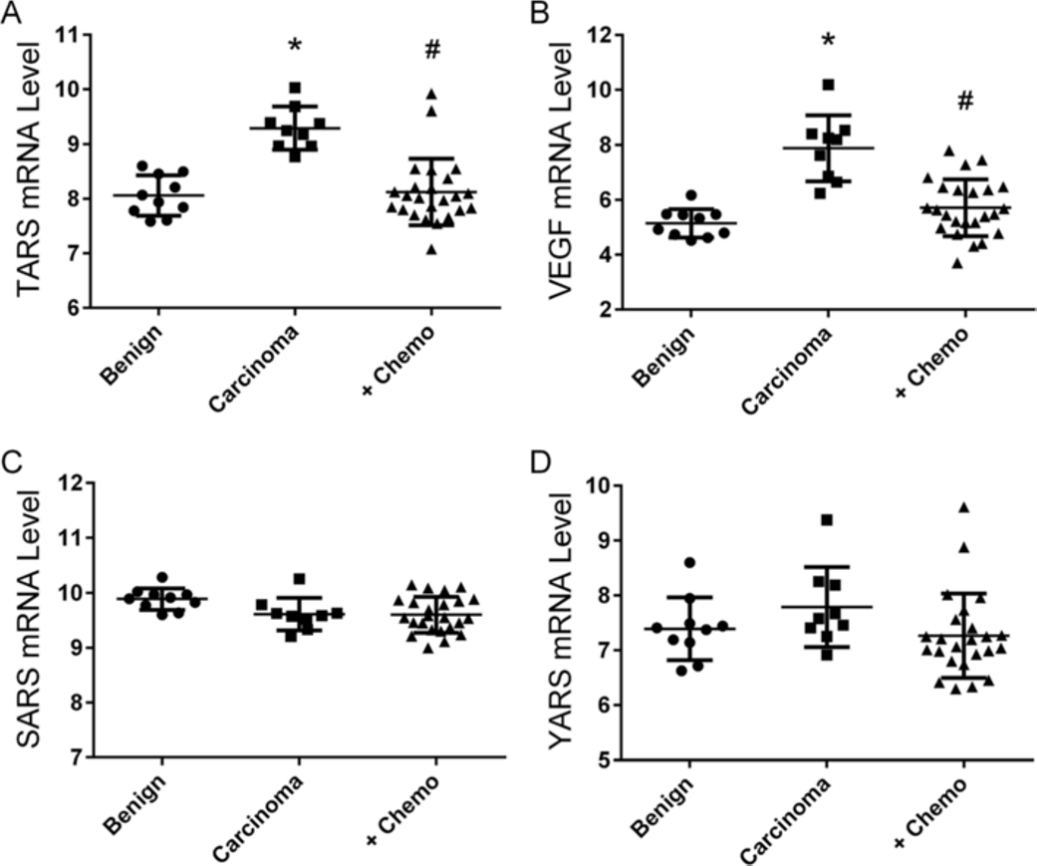
**TARS mRNA levels are increased in ovarian cancer and reduced by neo**-**adjuvant chemotherapy.** Data were obtained from GEO dataset GDS 2785. The study analyzed mRNA by GeneChip microarray in ovarian tissue from 10 patients with benign conditions, 9 patients with untreated adenocarcinoma, and 24 patients with adenocarcinoma treated with carboplatin/taxol prior to surgery (+Chemo) [18]. Shown are expression profile comparisons for **A**, TARS, **B**, VEGF, **C**, SARS and **D**, YARS. Differences between groups was significant for TARS and VEGF as determined by ANOVA Kruskal Wallis analysis, *p < 0.0001 compared with benign, #p < 0.001 compared with carcinoma.

To establish a connection between TARS and human ovarian cancer we first explored the available database resources for existing information. According to the CGAP mRNA database, TARS is over-expressed in select cancers including ovarian (56 tags) and colon adenocarcinoma (35 tags) [17]. In tissue arrays displayed in the Human Protein Atlas, TARS protein is moderately expressed in normal tissues but is highly expressed in ovarian tumors (9 of 12) [15]. Analysis of a GEO Profile dataset of ovarian cancer samples revealed that TARS mRNA levels are significantly upregulated in ovarian carcinoma and reduced in patients treated with neoadjuvant carboplatin/paclitaxel chemotherapy (Figure 1, also see Additional file 2) (GEO accession GDS2785; published by Moreno et al. [18]). The TARS levels also parallel the levels for VEGF in the same group of patients. This pattern of expression was not seen for seryl-tRNA synthetase (SARS) or tyrosyl-tRNA synthetase (YARS), suggesting that overexpression is not a characteristic of all amino-acyl tRNA synthetases (aaRS). These data provide support for investigating the role of non-canonical signaling by TARS in the angiogenesis and progression of ovarian cancer.

### TARS expression in patient tumor samples is positively correlated with stage of disease and angiogenic markers

**Figure 2:**
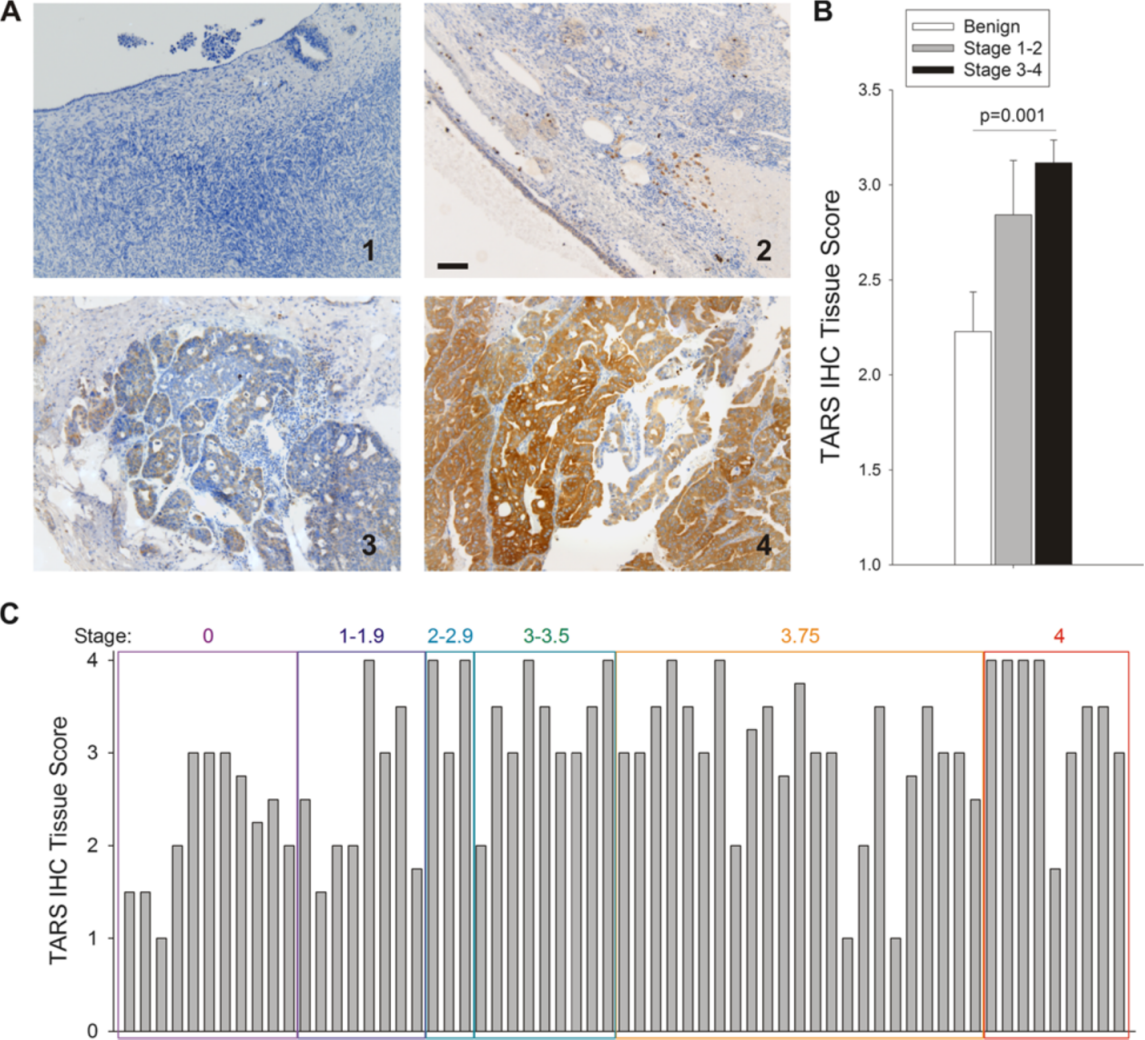
**TARS expression increases with stage of ovarian cancer. A**. Tissues were stained for TARS by immunohistochemistry and counter-stained with Mayers’ hematoxylin. Images were scored blindly by 2 independent investigators and tumor identification was later confirmed by a pathologist (SLM). Shown are 10x images from (1) normal ovary and examples of TARS score increasing with extent of disease (2–4), Bar = 100 μm. **B**. Graph representing average scores where high stage is ≥ stage 3; statistical significance determined by one-way ANOVA. For more detailed statistics, see Table 1 for Pearson’s Correlations. **C**. Shown is a graph of individual patient TARS tumor scores grouped according to FIGO stage of disease (i.e. Stage 3.75 = FIGO 3C).

**Figure 3:**
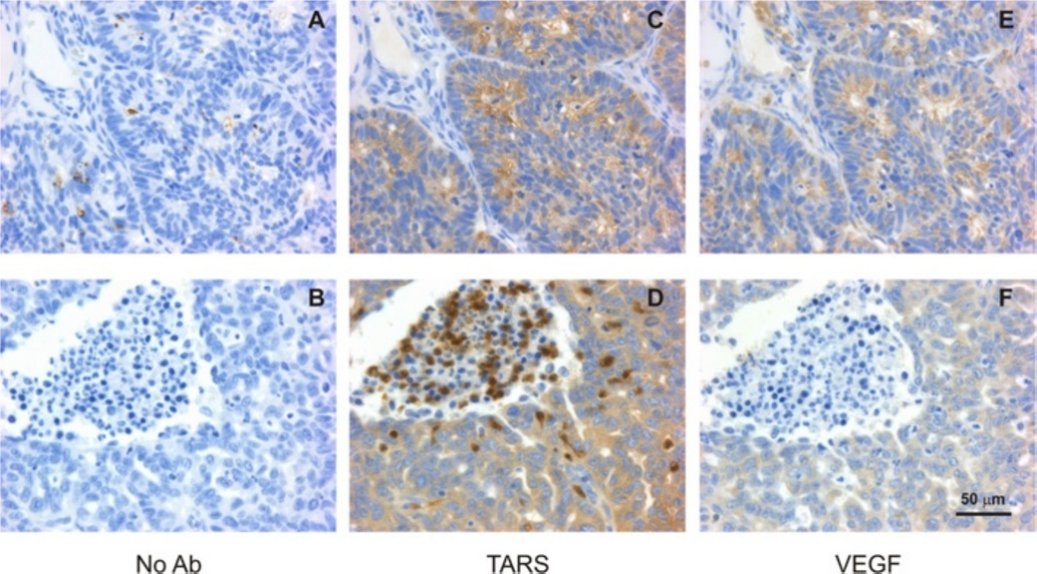
**TARS expression co** **-** **localized with VEGF and in leukocytes**. Tissues were stained by IHC as in Figure 2. Shown are 40x images of serial sections stained with **(A,B)** No primary Ab as a negative control **(C,D)** TARS or **(E,F)** VEGF. Bottom panels show examples of TARS staining in infiltrating leukocytes. Bar = 50 μm. See Additional File 3 for supporting images.

To validate the clinical links suggested by the database studies and the mechanistic links found in our angiogenesis study, we measured TARS expression in tissue sections from 70 human ovarian cancer specimens, of which 59 had 10 year patient survival information and 31 had matching pre-surgical serum samples [5]. Because TARS has a protein synthesis function in all cells, the antibody dilution for IHC was optimized to detect only overexpressed TARS. Of note, normal fallopian tube epithelium exhibited high TARS staining, but its significance relative to transformation was outside the scope of this study [19, 20]. Analysis by Pearson correlation matrix revealed a significant positive correlation (p < 0.001) between increasing disease stage and TARS staining intensity in ovarian tumor cells (Figure 2, Table 1). Overexpressed TARS also co-localized with VEGF (Figure 3) and was in proximity to areas of neovascularization indicated by the endothelial marker PECAM (CD31) (See Additional file 3A). These data reveal a novel link between TARS expression and ovarian cancer and confirm an association between TARS and angiogenic potential in the ovarian cancer microenvironment.

**Table 1:** **Pearson Correlation Matrix for TARS expression in ovarian cancer patient samples**

	Stage	TARS Tumor	VEGF Tumor	TARS Serum	TNF-α Serum
	r, *p*-*value*	r, *p*-*value*	r, *p*-*value*	r, *p*-*value*	r, *p*-*value*
TARS Tumor*	**0.436**, <***0.001***		**0.448**, <***0.001***	**0.455**, ***0.003***	0.129, *0.429*
**TARS Immune**	**0.296**, ***0.017***	**0.472**, <***0.001***	0.165, *0.188*	0.229, *0.167*	0.164, *0.325*
**TARS Serum**	0.233, *0.153*	**0.455**, ***0.003***	**0.298**, ***0.062***		0.155, 0.269

*Numbers represent the correlation (r) and significance (p-value) for each set of variables when compared with TARS (tumor and immune cells scores or serum pg/ml). Significant correlations are indicated in bold.

### TARS is overexpressed in infiltrating leukocytes within ovarian tumors

In the process of scoring the tumor tissue, we observed that many tumors (32 of 70) exhibited positive TARS staining in the cytoplasm of infiltrating leukocytes (Figure 3). These cells were histologically identified as neutrophils and plasma cells due to their chromatin characteristics (See Additional file 3B). A significant correlation was found between leukocyte TARS staining and both tumor TARS and stage of disease (p = 0.017), however the regression coefficients were weaker and the association with VEGF was not significant (Table 1). In light of the connection between TARS and immune responses, these data may indicate a role for TARS in the immune cell response to ovarian tumors.

### TARS is secreted from ovarian cancer cells in response to cell stress

**Figure 4:**
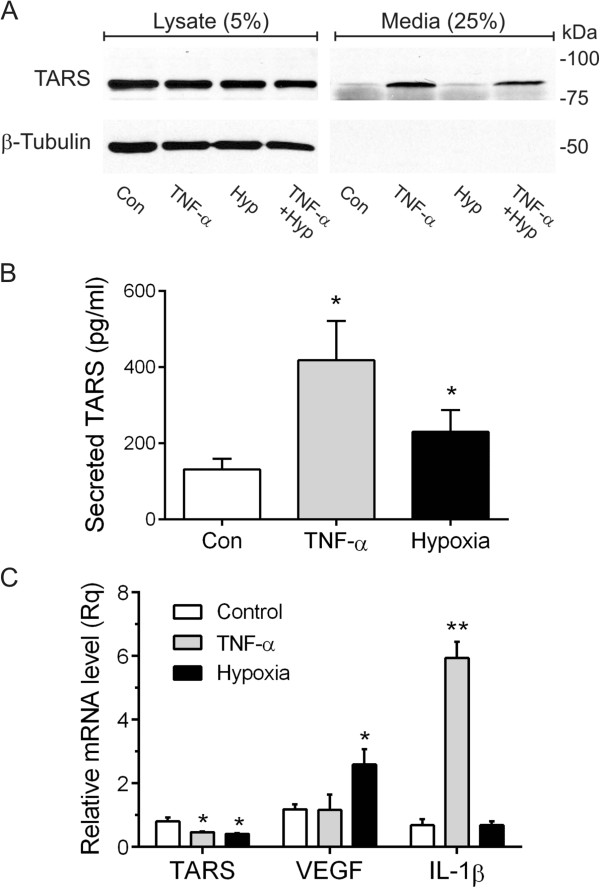
**TARS is secreted by SK** **-** **OV** **-** **3 ovarian cancer cells. (A)** SK-OV-3 cells were treated with TNF-α (50 ng/ml) or exposed to 2% O_2_ for 24 h where indicated. Media was concentrated 20-fold to accommodate 25% onto the gel and compared to 5% of the cell lysate. Shown is a representative Western blot probed for TARS and β-tubulin, n = 4. **(B)** Cells were treated as in **(A)**. After 24 h the level of TARS in the supernatant was determined by ELISA, *p < 0.05, n = 3. Cell membrane integrity was confirmed using the lactate dehydrogenase assay CytoTox-ONE™ and by lack of β-tubulin in media samples. **(C)** Relative mRNA levels for TARS, VEGF and IL-1β were determined by RT-qPCR following treatment as in **(A)**; *p < 0.001, **p < 0.0001, n = 4.

Our previous observation that extracellular TARS promotes endothelial cell migration and angiogenesis suggested that TARS may be secreted by ovarian cancer cells as a cell stress signaling response. The association between TARS autoantibodies and a TNF-α-related autoimmune disorder further supported this possibility. To measure TARS secretion, cultured human ovarian cancer cells (SK-OV-3) were incubated with TNF-α or exposed to hypoxia (2% O_2_), followed by assessment of TARS in the media using Western blot and a TARS-specific ELISA. As shown in Figure 4A,B, TNF-α promoted an increase in TARS in the cell media. The hypoxia response was more modest, but significant by ELISA. TARS was not present in the media due to cell lysis, as confirmed by a cytotoxicity assay and the lack of β-tubulin in the media samples. The increase in secretion was not a direct consequence of TARS transcription or mRNA stabilization since hypoxia and TNF-α treatments reduced TARS mRNA levels, whereas VEGF and IL-1β were increased respectively (Figure 4C). Taken together with our previous data, these findings suggest that ovarian cancer cells generate extracellular TARS as part of their cell stress signaling response.

### Serum levels of TARS correlate with tumor levels of TARS

**Figure 5:**
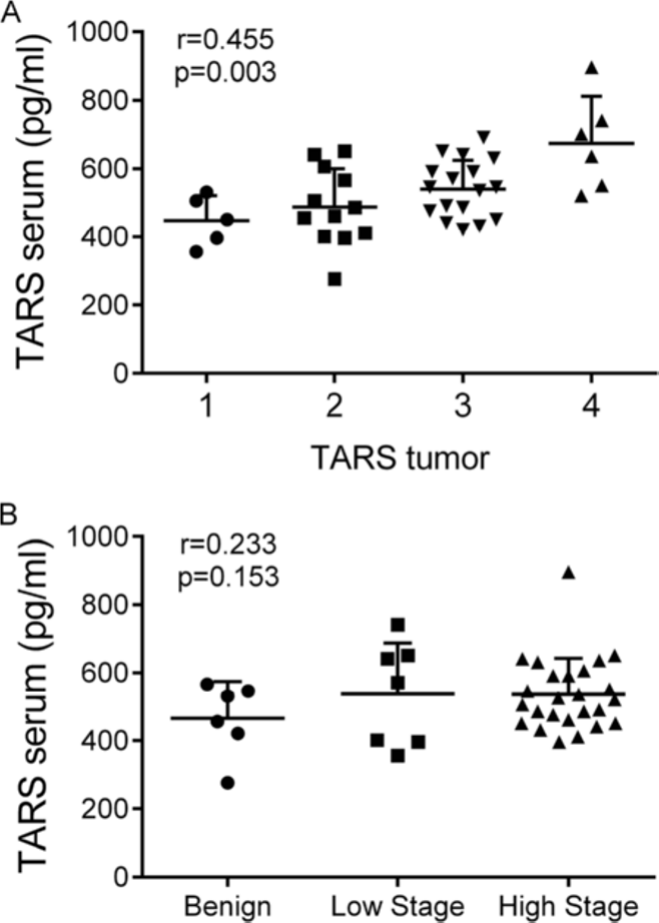
**Correlation of TARS levels detected in serum with TARS expression in ovarian cancer.** Graphs represent scatter dot plots (mean ± SD) of patient data for TARS serum levels against **(A)** Stage of cancer and **(B)** TARS tumor tissue score. The correlation (r-value) and p-values were calculated by a Pearson correlation matrix (see Table 1).

If the TARS level increases with stage of disease and it is secreted in response to cell stress, it is possible that TARS could provide value as a serum diagnostic for ovarian cancer. Serum samples were available for a subset of 6 controls and 31 patients with ovarian cancer that had matched tumor samples in the TARS IHC analysis. ELISA values were determined for TARS and TNF-α. Values for CA-125 were obtained from previous serological testing at diagnosis [21]. The TARS levels in patient serum samples ranged from 266–896 pg/ml. There was a significant correlation between the patient serum levels of TARS and the TARS tumor score, but levels did not correspondingly increase with stage of disease, (Figure 5, Table 1). These data suggest that TARS can be detected in serum, and encourage its further study as a potential indicator of ovarian cancer.

Because of the link between TNF-α and TARS secretion by cultured ovarian cancer cells as well as the TNF-α association with myositis, we predicted that the serum levels for TARS would parallel those for TNF-α. The TNF-α levels ranged from 0.145-12.0 pg/ml and levels did not correlate with stage of disease or level of TARS in tumors or serum (Table 1). When compared with other cancer patient studies, the levels of TNF-α in our study were lower in both controls and cancer patients [22]. Thus, although no correlations were found between TNF-α and TARS or ovarian cancer, more studies are needed using additional samples to account for variability and low levels of detection.

### Overexpression of TARS correlates with increased survival in late-stage disease

Cox proportional hazard models were used to assess the relationship between TARS expression and survival within the patient study group (Tables 2 and 3). Survival was measured in years alive after diagnosis (range 0.5-14 yrs). As expected there was an increase in mortality risk with stage of disease and serum levels of CA-125. The Hazard Ratio increased by 1.654 for each unit increase in stage. There was no significant survival relationship to serum levels of TNF-α or TARS. Surprisingly, TARS tumor expression was inversely related with mortality in advanced stage tumors as reflected by a negative trend in the regression coefficient (−0.394, p = 0.101). A multivariate Cox proportional hazard model was generated to adjust for Stage and year of diagnosis (Table 3). The number of patients and events in Stage 0, 1 and 2 was very low, thus they were grouped. This analysis strengthened the relationship between TARS and survival (−0.465, p = 0.062). Because adjustment for differing Stage groupings and diagnosis years did not impact the negative relationship between survival and TARS expression the results are not likely due an artifact of small sample sizes or patient population.

**Table 2:** **Hazard ratios for the association of TARS with mortality using univariate Cox proportional hazard models**

Parameter	n	Cox coefficient	SEM	Hazard Ratio	p-value
**Stage**	59	0.503	0.211	1.654	0.017
**CA** **-** **125**	35	0.412	0.155	1.510	0.008
**TNF**-**α** **(** **pg** **/** **ml** **)**	37	0.520	0.318	1.682	0.102
**TARS** **(** **pg** **/** **ml** **)**	37	0.281	1.272	1.324	0.825
**TARS tumor**	59	−0.394	0.240	0.674	0.101

**Table 3:** **Multivariate Cox proportional hazard model for TARS tumor score**

Variable	Cox coefficient	SEM	Hazard ratio (95% CI)	p-value
Stage grouping (0–2, 3, 4)	0.742	0.304	2.100 (1.161-3.80)	0.015
Year of Dx (1998–2001)	0.322	0.199	1.380 (0.936-2.03)	0.105
TARS tumor	−0.465	0.249	0.628 (0.387-1.02)	0.062

## Discussion

The goal of this study was to determine if there is a clinical relationship between TARS and the progression and angiogenic potential of human ovarian cancer. The data reveal the first positive correlation between TARS tumor expression and the progression of cancer. The overall results of the evaluation of TARS in tumor, immune cells, and serum provide strong support for a connection between TARS and both angiogenesis and stage of ovarian cancer. Furthermore, these data provide clinical relevance to our previous biologic observations of an angiogenic function of TARS. The observed secretion of TARS by ovarian cancer cells leads to a working model for TARS signaling that includes stress-mediated release of TARS that stimulates angiogenesis and alters the ovarian tumor microenvironment.

TARS is a member of the aaRS enzyme family whose primary function is to catalyze ATP-dependent formation of specific amino-acyl tRNAs, thus maintaining fidelity during protein translation [23]. Several aaRSs have been associated with non-canonical cell signaling related to inflammation, cell migration, and angiogenesis [24–30]. Those aaRSs associated with angiogenesis include tyrosyl-tRNA synthetase, which exhibits extracellular angiogenic activity, and seryl-tRNA synthetase, which acts in the nucleus to regulate VEGF expression during vascular development [26, 29]. Unlike TARS, these aaRSs were not upregulated in ovarian cancer according to the mRNA or protein database analyses. The selective association of TARS with ovarian cancer suggests that its extracellular activity may be specifically important in the regulation of the ovarian tumor microenvironment.

Angiogenesis has emerged as a critical component of the tumor microenvironment, and anti-angiogenic therapies for cancers have had success due to the reliance of many cancers on new vessels and the poor prognosis associated with cancers that have advanced angiogenesis [31]. Anti-angiogenic therapies for ovarian cancer have shown effectiveness in clinical trials using the VEGF inhibitor bevacizumab, especially when combined with the standard chemotherapy regimen (paclitaxel and carboplatin) [32]. Although these therapies have benefitted patients with ovarian cancer, treatment complications and lack of success in some patients demonstrate a clear need for more ovarian-specific angiogenic targets. We propose that TARS is an important angiogenic molecule that exhibits extracellular signaling in ovarian cancer cells only under conditions of metabolic stress. An agent that exclusively targets extracellular TARS thus has the potential to provide an anti-cancer therapy that is effective and more specific than existing treatments.

The finding that TARS is secreted by ovarian cancer cells in response to stress signaling is supported by the Exocarta proteomics database, where TARS is on the list of exosomal proteins released from bladder cancer cells, and ovarian cancer cells [33, 34]. An exosome mechanism for TARS secretion would be consistent with other signaling proteins that regulate the extracellular matrix and are overexpressed in cancer including heat shock protein 90 (HSP90), plasminogen activator inhibitor-1 (PAI-1) and caveolin-1 (cav-1) [35–37]. Although the correlation between serum TARS and stage of disease failed to reach significance, its presence at detectable levels and trend with TARS tumor levels indicate that it has potential value as an ovarian cancer diagnostic.

Two unexpected discoveries were the findings that TARS is overexpressed in infiltrating leukocytes within ovarian tumors and that mortality risk is slightly reduced for patients with high expression of TARS within their tumors. TARS has previously been associated with autoimmune disorders because it is the target for the autoantibody PL-7 in myositis disorders [12]. A specific role for TARS in immune cell signaling has not been reported, however several other aaRS enzymes exert immune cell functions [10] and TARS is among B cell proteins secreted in exosomes [38]. It is possible that the improved survival associated with TARS expression is related to its effects on inflammatory cell signaling that reduce the support of the microenvironment. This possibility is supported by evidence that infiltrating immune cells in the context of high vascularity improves survival in high grade serous ovarian cancer [39]. Alternatively, the signaling through TARS may improve the susceptibility of tumors to chemotherapy, similar to the improved response of ovarian tumors expressing p53 mutations [18]. The antigenic properties of TARS and the known role for immune cell signaling in angiogenesis encourage future study of TARS in immune cell responses to ovarian tumors.

## Conclusions

Taken together, the results of this study demonstrate that TARS expression is significantly increased in human epithelial ovarian cancer and relates to angiogenic markers and stage of disease. Opposing correlations of TARS with stage vs. survival suggest a complex role for TARS in tumor progression. We propose that TARS is secreted by tumor cells in response to metabolic stress or cytokine signaling, which then affects the microenvironment through vascular endothelial and immune cell responses. The limitations of this study include the number of patient samples and the IHC scoring technique. These weaknesses are balanced by the strength of the information for each patient and the ability to distinguish tumor staining within sections. The selectivity of TARS overexpression in ovarian cancer and its presence in patient serum samples encourages further study of TARS as a diagnostic or therapeutic target.

## Electronic supplementary material

Additional file 1: **Ovarian Cancer Patient Information.** (PDF 13 KB)

Additional file 2: **Correlative mRNA levels of TARS and VEGF in ovarian cancer patients.** (PDF 149 KB)

Additional file 3: **Images to supplement Figure** 3 **.** (PDF 608 KB)

## Abbreviations

TARS: Threonyl-tRNA synthetase
VEGF: Vascular endothelial growth factor
PECAM: Platelet endothelial cell adhesion molecule
CA-125: Cancer antigen-125
ELISA: Enzyme-linked immunosorbent assay
HIF-1α: Hypoxia inducible factor
IHC: Immunohistochemistry
TNF-α: Tumor necrosis factor-α
IL-1β: Interleukin-1β
aaRS: amino-acyl tRNA synthetase
SARS: Seryl-tRNA synthetase
YARS: Tyrosyl-tRNA synthetase.
