# Supplementary material for: Threonyl-tRNA synthetase overexpression correlates with angiogenic markers and progression of human ovarian cancer
Source: BMC Cancer. 2014 Aug 27;14:620. doi: 10.1186/1471-2407-14-620 (PMC4155084; doi:10.1186/1471-2407-14-620)
Supplement: Supplementary file 1 — Additional file 1: Ovarian Cancer Patient Information. (PDF 13 KB) [file 12885_2014_4804_MOESM1_ESM.pdf]

Additional File 1: Ovarian Cancer Patient Information

Table S1A: Ovarian Cancer Patient Group:

Tumor Samples

Patients	Control	Study
Number	12	70
Age Range (yr)	28-67	39-74
Pathology		
**Benign Conditions**	**12**	
**Papillary Serous Adenocarcinoma**		**59**
Stage I-II		6
Stage III		39
Stage IV		14
**Endometrioid Adenocarcinoma**		**7**
Stage I-II		3
Stage III		4
Stage IV		0
**Clear Cell Adenocarcinoma**		**4**
Stage I-II		3
Stage III		1
Stage IV		0

Table S1B: Ovarian Cancer Patient Group:

Serum Samples Subset

Patients	Control	Study
Number	6	31
Age Range (yr)	49-59	39-74
Pathology		
**Benign Conditions**	**6**	
**Papillary Serous Adenocarcinoma**		**25**
Stage I-II		4
Stage III		16
Stage IV		5
**Endometrioid Adenocarcinoma**		**3**
Stage I-II		1
Stage III		2
Stage IV		0
**Clear Cell Adenocarcinoma**		**3**
Stage I-II		2
Stage III		1
Stage IV		0
