# Supplementary material for: Threonyl-tRNA synthetase overexpression correlates with angiogenic markers and progression of human ovarian cancer
Source: BMC Cancer. 2014 Aug 27;14:620. doi: 10.1186/1471-2407-14-620 (PMC4155084; doi:10.1186/1471-2407-14-620)
Supplement: Supplementary file 2 — Additional file 2: Correlative mRNA levels of TARS and VEGF in ovarian cancer patients. (PDF 149 KB) [file 12885_2014_4804_MOESM2_ESM.pdf]

Additional File 2: Correlative mRNA levels of TARS and VEGF in ovarian cancer patients

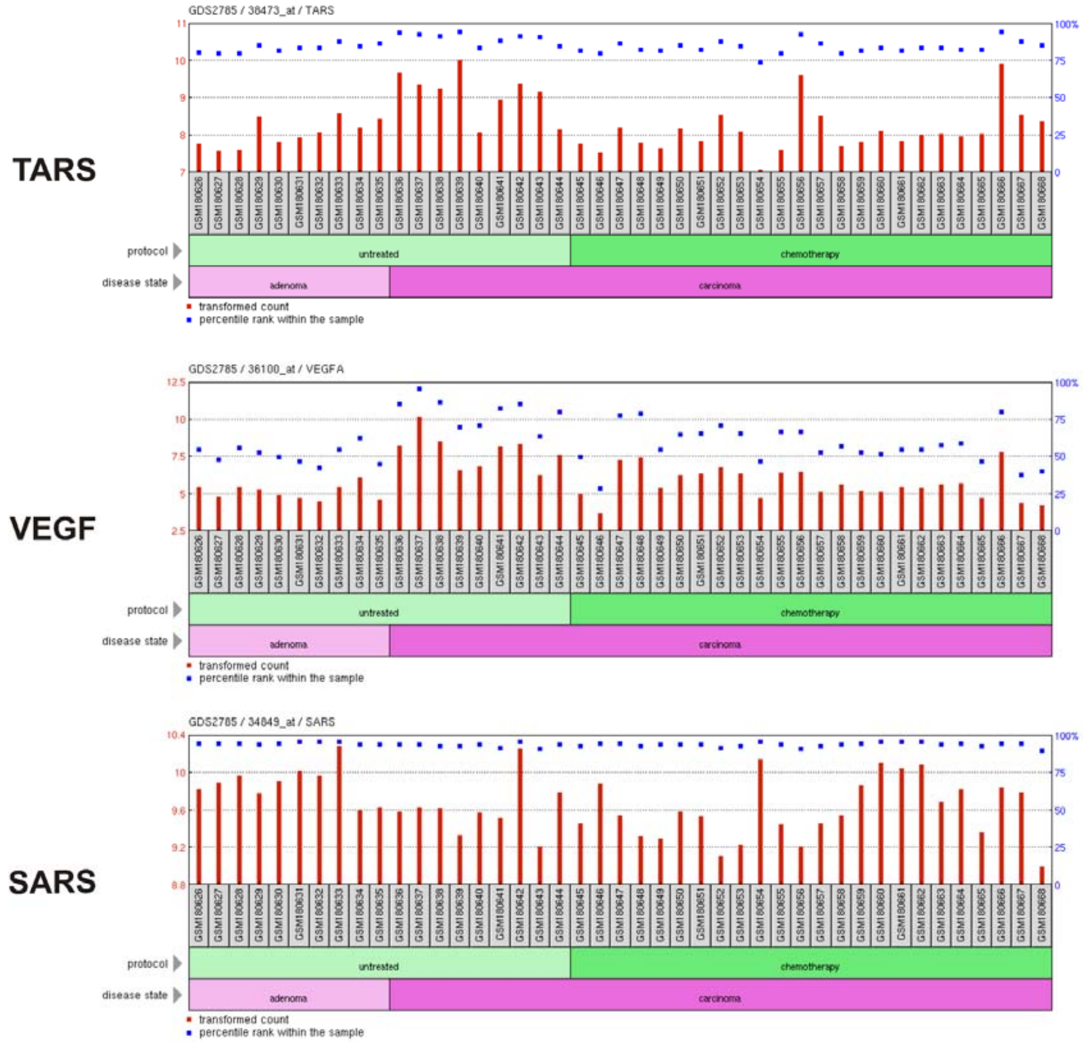

Shown are GEO datasets representing mRNA GeneChip expression profiles of TARS, VEGF and SARS in ovarian cancer patients. Patient categories included untreated adenoma (benign conditions), untreated carcinoma (papillary serous adenocarcinoma), and chemotherapy carcinoma (patients treated with carboplatin/paclitaxel therapy prior to surgery). Data were obtained from GEO accession GDS2785. Analysis of the raw data is shown in **Fig. 1**.
