# Supplementary material for: Threonyl-tRNA synthetase overexpression correlates with angiogenic markers and progression of human ovarian cancer
Source: BMC Cancer. 2014 Aug 27;14:620. doi: 10.1186/1471-2407-14-620 (PMC4155084; doi:10.1186/1471-2407-14-620)
Supplement: Supplementary file 3 — Additional file 3: Images to supplement Figure 3 . (PDF 608 KB) [file 12885_2014_4804_MOESM3_ESM.pdf]

Additional File 3: Images to Supplement Fig 3

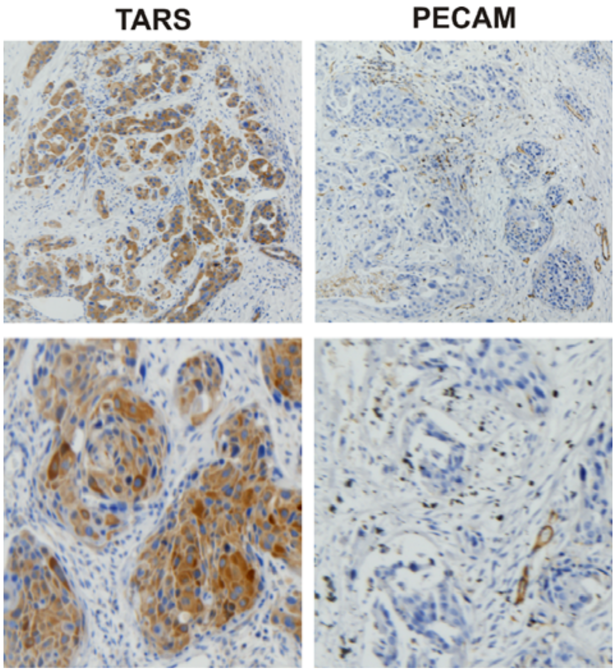

A. TARS expression correlates with neovasculature. IHC images showing co-localized TARS with microvascular endothelial cell PECAM staining in ovarian tumor sections magnified at 10x (top) or 40x (bottom).

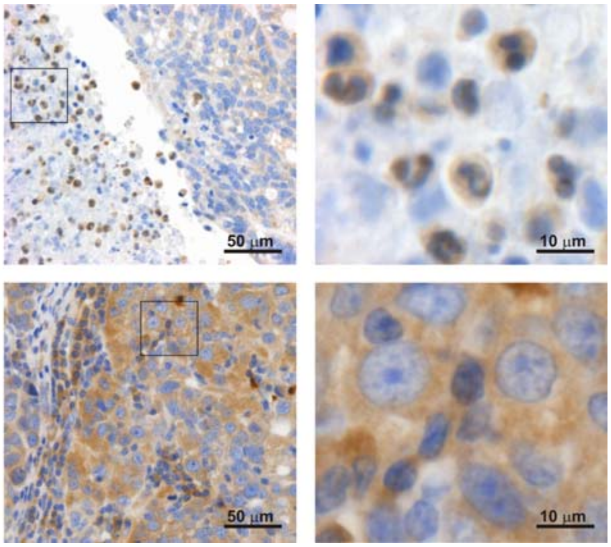

B. TARS expressed in infiltrating leukocytes within ovarian tumors. Shown are example IHC images from serial ovarian tumor sections (40x) stained with TARS that exhibit infiltrating leukocytes representative of neutrophils (top) and plasma cells (bottom). Box indicates magnified region shown in right panels.
